# Pulmonary Arteriovenous Malformation Unmasked by Pregnancy: A Review of Pulmonary Arteriovenous Malformations and Cardiovascular and Respiratory Changes in Pregnancy

**DOI:** 10.1155/2023/5469592

**Published:** 2023-03-28

**Authors:** Anita Lukic, Larisa Cmelak, Dominik Draženović, Hrvoje Kojundzic, Ivan Kresimir Lukic, Vicko Gluncic

**Affiliations:** ^1^Varazdin General Hospital, Varaždin, Croatia; ^2^Bjelovar University of Applied Sciences, Bjelovar, Croatia; ^3^Jordanovac Department of Thoracic Surgery, Zagreb University Hospital Centre, Zagreb, Croatia; ^4^Catholic University of Croatia, School of Medicine, Zagreb, Croatia; ^5^Department of Anesthesia, Mount Sinai Hospital, Chicago, USA

## Abstract

Pulmonary arteriovenous malformations are abnormal, direct communications between the branches of the pulmonary artery and pulmonary veins, but without pulmonary capillaries between them. During pregnancy, PAVMs can enlarge and become symptomatic, causing even serious complications like haematothorax. To recognize the PAVM that becomes symptomatic in pregnancy, one must be able to distinguish the patient's symptoms caused by developing complications of PAVM, as in the case we present, from physiological changes accompanying a healthy pregnancy, including their degree in relation to the stage of pregnancy. The modified early obstetric warning score charts are a very helpful tool in the assessment of (ab)normal signs and symptoms in pregnant women, especially for physicians who rarely manage pregnant women.

## 1. Introduction

Pulmonary arteriovenous malformations (PAVMs) are abnormal, direct communications between the pulmonary artery branches and pulmonary veins, but without pulmonary capillaries between them. They are the leading anomalies of the pulmonary vessels, usually congenital and without malignant potential [1, 2].

PAVMs can occur congenitally (>80%, mutations located on chromosome 12 or 9) or could be acquired later in life (<20%) [3, 4].

In 47-80% of cases congenital PAVMs are found associated with the autosomal dominant disease hereditary haemorrhagic telangiectasia, HHT (Osler-Weber-Render disease) [5], and it is believed that 5-35% of the population with HHT have a PAVM [5, 6].

The incidence of PAVM is 2–3 per 100 000 population [7], and they were described in patients of both genders (with a male-to-female ratio from 1 : 1.5 to 1 : 1.8) [8]. Although PAVMs can be diagnosed at any age, they are mostly discovered by the age of 30 years [3].

Usually, PAVMs occur in solitary (64%), while 36% of patients have multiple lesions, which often occur bilaterally [9]. Solitary PAVMs are most commonly seen in the left lower lobe, located subpleurally or in the outer third of lung parenchyma; they are usually 1-5 cm in size, but can also be microscopic or as big as 10 cm [3, 5, 10, 11].

## 2. Pathology and Pathophysiology of PAVM

PAVM can be simple (80% of cases have a single feeding segmental artery leading to a single draining pulmonary vein) or complex (20% of cases have ≥2 feeding arteries or draining veins) [12, 13]. They are classified into five groups (revised classification) based on the embryological development of the lung and pulmonary vasculature, Table 1 [3, 14].

The most prominent pathophysiological feature in PAVM is an elevated proportion of right-to-left shunt from the pulmonary artery to the pulmonary vein (in 88-100% of patients) [5, 15, 16]. Since PAVMs do not affect cardiac haemodynamic [16], electrocardiogram, blood pressure, cardiac haemodynamic indices, and pulmonary capillary wedge pressure are usually normal [3].

## 3. Clinical Findings in PAVM

PAVMs are asymptomatic in 13-55% of patients [17–19], while the development and amount/severity of symptoms are proportional to the size/number of PAVM and shunt; the patients with a single PAVM smaller than 2 cm or with a shunt <20% are usually asymptomatic [5].

On the other hand, in a detailed examination, abnormal findings could be found in as high as 75% of patients [20]. The most common symptom is dyspnea on excretion (in 31-67% of cases) [1, 5], the severity of which depends on the degree of right-to-left shunt and hypoxaemia. The SpO_2_ is decreased and does not normalise with 100% oxygen supplementation, but most patients do not experience symptoms until PaO2 is <8.0 kPa.

## 4. Diagnosis of Pulmonary Arteriovenous Malformations

PAVM should be suspected in clinical findings such as unexpected dyspnea, hemoptysis, hypoxemia, polycythemia, clubbing, cyanosis, mucocutaneous telangiectases, one or more pulmonary nodules on chest radiography, or incidents such as cerebral embolism or brain abscess [18].

Since 98% of patients show abnormalities in a plain chest radiograph (round or oval sharply defined masses of uniform density, frequently lobulated, from 1–5 cm in diameter in size) [3, 5, 10, 16], chest radiography is an important step for both the diagnosis and the further follow-up of PAVM.

Although the vascular anatomy of a PAVM could be studied by selective pulmonary angiography, contrast-enhanced computed tomography is better in detecting a PAVM (98 vs. 60%), including multiple small PAVMs [21], while angiography is superior in determining the vascular architecture of individual PAVMs [21].

The gold standard for the diagnosis of PAVM is pulmonary angiography [1, 16, 21], and it is used both to diagnose PAVMs (including the detection of unsuspected PAVM within the thorax and extrathoracically) and to determine the vascular architecture of individual PAVMs. It is especially valuable tool in planning further therapy. Also, PAVMs could be evaluated by three-dimensional (3D) helical computed tomography [21] and magnetic resonance imaging [22], although both methods have their limitations [21, 22].

The presence of the right-to-left shunt due to PAVM is the most easily detected by contrast echocardiography; agitated saline or dye is injected into a peripheral vein, and the air bubbles appear in the left atrium with a delay of three to eight cardiac cycles without being trapped in the pulmonary circulation [23]. Although contrast echocardiography is extremely sensitive in detectiong left-to-right shunt it cannot assess the amount of the shunt quantitatively. On the other hand, it is useful for the assessment of the efficiency of embolotherapy [24].

The amount of right-to-left shunt in PAVM could be evaluated by measuring the oxygen saturation and arterial oxygen pressure after breathing 100% oxygen for 15 to 20 minutes [3, 23] or by radionuclide perfusion lung scanning [3, 24].

To sum up, the reasonable algorithm for the diagnosis of PAVM could be the contrast-enhanced computed tomography following an abnormal chest radiography and additionally supplemented by contrast echocardiography to determine the presence of a right-to-left shunt, and the shunt fraction is determined by the 100% oxygen method. If any intervention is planned, pulmonary angiography is advised to explore the anatomy of the PAVM.

## 5. Treatment of PAVMs

Since the morbidity is higher in untreated than in treated patients (50% in untreated vs. 3% in treated patients) [5, 20], and due to the fact that all PAVMs enlarge over time and that the enlargement of the PAVM is more prominent in untreated PAVMs [1, 5, 24], treatment is offered to all patients with PAVMs larger than 3 mm, regardless of clinical symptoms [25].

The two main treatment options for PAVM are surgical resection (in various extents and surgical approaches) and percutaneous endovascular embolotherapy of the feeding vessel by coils or plugs/balloons (treatment of choice), with each method bearing some advantages, disadvantages, and complications [26].

## 6. The Case Presentation

The 26-year-old patient, without prior health issues, presented herself for the first time to the clinical emergency department (ED) at 20 weeks of pregnancy due to the sharp pain on the left side of the chest that radiated under the left scapula, in the left side of the neck, and in the left shoulder. The pain got intense when she moved her torso or left arm and during the deep inspiration. That day, the pain started three hours prior to her presentation in the ED, but she had the same pain on two occasions during the previous week, but they were much less intense. Physical findings (including breathing sounds), ECG, and laboratory findings were normal [including hemoglobin level (128 g/L)], except leukocytes of 1.5 times the normal range (16.6 × 10^9^/L), with 85% of neutrophils, and elevated D-dimer levels (two times the normal level, 1193 ng/mL). The chest radiography was not done due to the pregnancy. Gynecological exams, as well as ultrasounds, were normal. Later that day, heart ultrasound, ambulatory continuous 24-hour blood pressure monitoring, and continuous 24-hour ECG Holter monitoring were done, and all were within normal limits.

A month later (24 weeks of pregnancy), the ambulance was called to her home because she had pain in her whole left chest and she felt dyspnea because the pain limited full inspiration, but she did not meet the criteria for hospitalization (BP 110/60 mmHg, pulse 100/min, SpO2 97%, normal physical status, including breathing sounds). She was instructed to take paracetamol. The next day, she came to the ED for the second time, complaining of chest pain, frequent epistaxis, and dyspnea on exertion. The physical examination (including breathing sounds) and ECG was normal; laboratory findings were normal, except for the erythrocytes (2.99 × 10^12^/L), hemoglobin level (99 g/L), haematocrit (0.28), leukocytes of 1.5 times the normal range (15.5 × 10^9^/L), with 81% of neutrophils, and elevated D-dimer levels (two times the normal, 1370 ng/mL), as a month before. In acid-base status, PaO_2_ was 8.84 kPa, while PaCO_2_ was 3.5 kPa. Again, the chest radiography was not done due to the pregnancy.

Ten days later (25^+4^ weeks of pregnancy), the patient was referred to the ED by a gynecologist due to dyspnea and left chest pain on deep inspiration (the gynecological exam was normal). On physical examination, BP was 120/60 mmHg, pulse 112/min, SpO2 96%, and 20 breaths/min, with decreased breath sounds on the left chest. ECG were normal, and laboratory findings were normal, except for the erythrocytes (2.7 × 10^12^/L), hemoglobin level (87 g/L), haematocrit (0.26), leukocytes of 1.5 times the normal range (15.3 × 10^9^/L), with 79% of neutrophils, and very elevated D-dimer levels (13256 ng/mL), with almost normal acid-base status (PaO_2_ was 11.9 kPa, while PaCO_2_ was 3.3 kPa). The heart ultrasound was normal.

**Figure 1:**
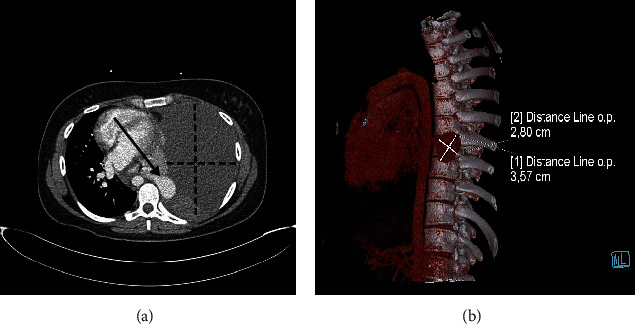
Computed tomography pulmonary angiography at 25^+4^ weeks of pregnancy of the patient presented with dyspnea on multiple occasions from 20 weeks of pregnancy. (a) Transversal plane, arrowhead pointing at arteriovenous malformation, and dotted lines indicating diameters of left haemathothorx; (b) isolated mass—arteriovenous malformation.

Because pulmonary embolism was suspected, CT pulmonary angiography was performed after the situation was explained to the patient, and an obstetrician was consulted. The CT pulmonary angiography revealed no pulmonary embolism, but evealed collapsed left lung, and the shift of mediastinal structures to the right side, with massive pulmonary pleural effusion on the left side with high densities suggesting hematothorax, (Figure 1(a)). Also, there was a paravertebral hyperdense zone of active bleeding (Figures 1(a) and 1(b)).

The patient was transferred to the surgical intensive care unit (ICU), transfused with 750 mL of erythrocyte concentrate, and prepared for the left thoracic drainage under total intravenous anaesthesia. The anaesthesia and the placement of the thoracic tube went uneventful, and 1500 mL of haematic effusion was drained immediately after the introduction of the chest tube.

**Figure 2:**
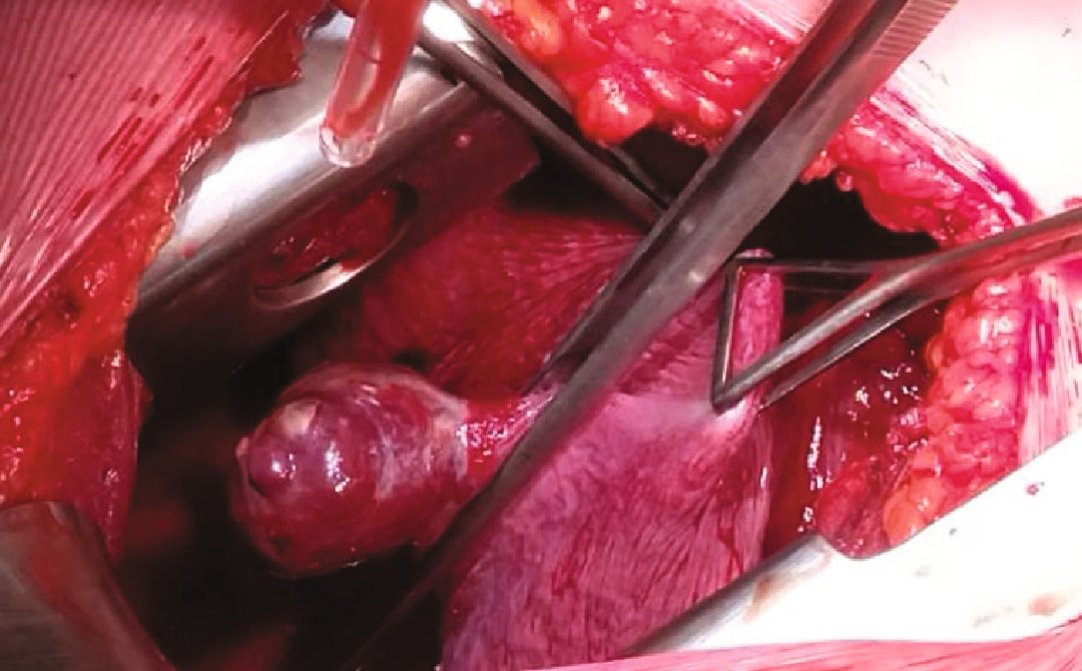
Intraoperative finding at 25^+4^ weeks of pregnancy of the patient presented with dyspnea on multiple occasions from 20 weeks of pregnancy. Computed tomography pulmonary angiography at 24 weeks of pregnancy revealed isolated arteriovenous malformation.

After the initial stabilization of the patient, she was transferred to a tertiary thoracic surgery center, accompanied by an anaesthesia team, where she was operated in the same afternoon. A left thoracotomia was done in the left lateral position, with the evacuation of haematoma and the atypical resection of the inferior lobe of the left lung due to the mass that appeared as a pulmonary arteriovenous malformation (Figure 2). The patient was stable, and the anaesthesia was uneventful.

The early and late postoperative courses were uneventful, and the following multiple gynecological examinations were normal. The elective cesarean section was performed in 38^+4^ weeks of pregnancy due to the previous pulmonary operation, gestational diabetes, and local obstetric findings.

**Figure 3:**
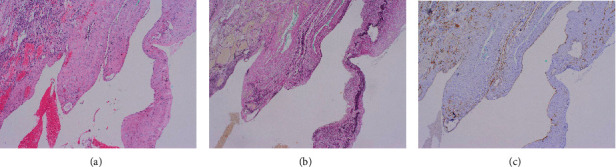
Microscopic appearance of mass removed from the lung of the patient at 25^+4^ weeks of pregnancy presented with dyspnea on multiple occasions from 20 weeks of pregnancy. (a) Hematoxylin-eosin staining, original magnification ×10; (b) ERG staining, original magnification ×10; (c) CD34 staining, original magnification ×10.

Pathohistological analysis of the mass showed lung parenchyma with collapsed alveoli and vascular structures with endothelial cells (CD34, CD31, and ERG positive), and partially organized thrombi, indicating a possible arteriovenous malformation (Figure 3).

During her follow-up examinations, the patient was advised to do screening for HHT; MR angiography of the brain and abdomen revealed no additional AV malformation, but three additional PAVMs were found, while the genetic analysis is not done yet.

## 7. Discussion

Our patient presented herself in the clinical ED with persistent chest pain and dyspnea on exertion, which is one of the three most common symptoms of PAVM [1, 5, 7]. Unfortunately, the fact that she was pregnant delayed the correct diagnosis in two ways: (1) her symptoms were wrongly attributed to the pregnancy, and (2) chest radiography—the first-line diagnostic test—was not done due to the pregnancy.

Although our patient had symptoms for more than a month before the diagnosis, her symptoms (dyspnea and chest pain) were repeatedly attributed to the pregnancy by different physicians after the coronary incident was ruled out each time. However, the fact remains that pregnancy is usually not associated with chest pain of that sort or this level of dyspnea, especially at that stage of pregnancy (20-25 weeks of pregnancy), which had to be taken into account.

## 8. Physiological Changes in Pregnancy

It is true that during pregnancy, manly progesterone and estrogen mediate anatomical and physiological changes by direct or indirect influence, with the cardiovascular (Table 2) and respiratory systems (Table 3) being affected the most (except for the genital system). Because of these changes in physiology and anatomy, assessing the physical status of a pregnant woman and interpreting signs, symptoms, and laboratory findings can be challenging. Therefore, to accurately assess the deviations from pregnant-normal, it is important to know when and to what degree of physiological changes during the pregnancy occur. The importance of this is well recognized and acknowledged, so the modified early obstetric warning score charts (MEOWS charts) were developed for the timely recognition, treatment, and referral of women who might develop a critical illness during or after pregnancy [27].

Knowing the usual degree of changes in pregnancy for the given stage of pregnancy and taking them into account, along with using the MEOWS chart, would raise suspicion that something is not physiological much earlier, as in our patient, and probably stern the diagnosis into the right direction, and the chest radiography would be advised in her earlier visits to the ED.

Since PAVMs are usually congenital, and our patient's medical history did not suggest that her PAVM was acquired [4, 5, 16], we reckon that she had an asymptomatic PAVM earlier in life, but during the pregnancy, it became symptomatic. Namely, during pregnancy, PAVMs enlarge due to the increased pulmonary blood flow caused by increased blood volume and cardiac output that accompany pregnancy (and are mediated by pregnancy hormones) (Table 2) [17]. There are two main mechanisms for enlargement: (1) Since the vascular resistance in the PAVM is lower than in the normal artery-capillary-vein circulatory system, blood preferentially flows through the low resistance PAVM [17], and the increased blood flow across the PAVM causes its dilatation. (2) Furthermore, high levels of progesterone accompanying the pregnancy cause vasodilatation, including of pulmonary vessels, that leads to an additional increase of blood flow and enlargement of the PAVM [17]. Such enlargement of PAVMs could be associated with more frequent complications [17, 19], such as spontaneous haemothorax following intrapleural rupture of PAVM [17, 19], as in our patient, or even the risk of maternal death in 1% of cases [4].

The other quite possible diagnosis in our patient was pulmonary thromboembolism (PTE) since the patient had chest pain, dyspnea, and elevated D-dimer levels which are the leading symptoms of PTE. Since PTE is not uncommon in the second trimester [46], a CT pulmonary angiography was done. Although it ruled out the PTE, it revealed a hematothorax, collapsed lung, and the shift of mediastinal structures to the right side, which eventually led to the correct diagnosis of PAVM.

Except for the fact that the symptoms were repeatedly attributed to the pregnancy instead of the pulmonary disease, the reluctance to do chest radiography also contributed to the delayed diagnosis. Although the risk of radiation exposure is a frequent concern when radiographs are considered when diagnosing a pregnant patient, it is well known that the devastating effects of radiation exposure in fetuses are seen with high radiation doses and that the risk of radiation exposure decreases with gestational age [4]. Moreover, it is even recommended that from early in the second trimester, radiologically guided PAVM embolization could be done, since at this stage of fetal development there is the least fetal susceptibility to radiation [4]. In addition, inviting an obstetrician early in the assessment of a pregnant patient presenting with dyspnea, rather than simply dividing roles depending on whether it would be an obstetric or an internal problem, would largely eliminate the delay in diagnosis since the obstetrician would probably not avoid taking a chest X-ray in the situation of the 20th week of pregnancy. Therefore, we advocate the importance of critical appraisal of radiology imaging when needed despite the pregnancy and include the obstetrician early in the process so that the diagnosis would not be delayed.

## 9. Conclusion

So, just as some other diseases can be undisguised in pregnancy, e.g., diabetes or hypertension, PAVM can reveal itself during the course of pregnancy due to physiological changes.

Therefore, it is important to bear in mind that a pregnant woman can still suffer from other diseases that are not related to pregnancy, especially if the severity of symptoms is not correlated with the stage of pregnancy. In light of that, this case report emphasizes that when assessing the status, symptoms, and signs of a pregnant woman, one must have knowledge of the degree of physiological changes in pregnancy. Additionally, using the MEOWS chart could guide staff in monitoring a pregnant woman and early recognition of signs and symptoms that are not within the range of normal physiological changes associated with pregnancy.

## Figures and Tables

**Table 1:** Classification of pulmonary arteriovenous malformations [3, 14].

Group	Subgroup	Features
I		Multiple small arteriovenous fistulas
		Without aneurysm

II		Large arteriovenous aneurysm

III	A.	Large arteriovenous aneurysm (central)
	B.	Large arteriovenous aneurysm with anomalous venous drainage
	C.	Multiple small arteriovenous fistulas with anomalous venous drainage

IV	A.	Large venous aneurysm with systemic artery communication
	B.	Large venous aneurysm without fistula

V		Anomalous venous drainage with fistulas

**Table 2:** Physiological changes of the cardiovascular system during the pregnancy [28–38].

Feature	Change	Week of pregnancy/trimester
Blood volume	↑ 1-2 L (up to 40-50%)	Increase from 7th week, peaks at 30-34 weeks
Plasma volume	↑ By 30–50% (=1200-1600 mL) till 70% in twin pregnancies	
Plasma colloid osmotic pressure	↓ Of 10–15%	
Colloid osmotic pressure/pulmonary capillary wedge pressure gradient	↓ By 30%	
Total body water content	↑ By 6.5-8 L	
Red blood cells	↑ By 18-25%	
Haematocrit	↓ To 32-34%	
Platelet count	↓ By 20%	By term
PT, aPTT	Shortened	By term
Factors II and V, protein C	No change	By term
Fibrinogen, factors VII, VIII, IX, X, XII, and VWF	↑ More than 100%	By term
Factor XI	Variable	By term
Factor XIII, protein S	↓	By term
D-dimer	↑ Up to 400%	By term
Preload	↑	
Afterload	↓	
Cardiac output	↑ To 30-50%	From 6th week, peak between 16 and 28 weeks, decreases slightly from 30 weeks until labor
Stroke volume	↑ By 23-30%	
SVR	↓ By 20%	Nadir at 20 week
	↑	Gradual rise after 20 weeks, till term
Heart rate	↑, Generally not above 100 beats/minute	
Systolic blood pressure	=/↓ By 10-15 mmHg	In 1st trimester, returns to baseline in the second half of pregnancy
Diastolic blood pressure	↓ By 10-15 mmHg	Nadir at 28 week, returns to baseline in the second half of pregnancy
	↑	Gradual rise, till term
Pulmonary circulation	↑	
Pulmonary capillary wedge pressure	=	
Pulmonary vascular resistance	↓	

Abbreviations: PT: prothrombine time; aPTT: activated partial prothrombine time; SVR: systemic vascular resistance.

**Table 3:** Physiological changes of respiratory system during the pregnancy [37, 39–45].

Feature	Change	Week of pregnancy/trimester
Chest compliance	↓	
Total lung capacity	↓ By 5%	
Vital capacity	=	
The functional residual capacity	↓ By 10-25%	
Functional residual capacity	↓ By 10-25%	
Expiratory reserve volume	↓ By 15-20%	
Tidal volume	↑ By 30-50% (from 500 to 700 mL)	
Minute volume	↑ By 40%	During 1st trimester
Respiratory rate	↑ By 1-2 breaths more than normal	
Minute ventilation	↑ By 20–50% (from 7.5 to 10.5 L/min)	
Residual volume	↓ By 20-25%	
The expiratory reserve volume	↓ By 15–20% (200-300 mL)	
The residual volume	↓ by 20–25% (200-400 mL)	
Total lung capacity	↓	
Respiratory capacity	↑ By 5-10%	
Inspiratory capacity	↑ By 5–10% (200–350 mL)	
The total lung capacity	↓ By minimally 5%	By term
Spirometry parameters (FVC, FEV1)	=	
Oxygen consumption	↑ By 20-40%	
Metabolic rate	↑ By 15%	
Oxygen reservoir	↓	
PaO_2_	↑	
PaCO_2_	↓	
Bicarbonate levels	↓ To 18–21 mmol/L	
Blood pH	↑ To 7.44 (respiratory alkalosis with a compensatory metabolic acidosis)	

Abbreviations: FVC: forced vital capacity; FEV1: forced expiratory volume in 1 second; PaO_2_: partial pressure of oxygen in the arterial blood; PaCO_2_: partial pressure of carbon dioxide in the arterial blood.

## Data Availability

The data (clinical/laboratory findings and medical knowledge) used to support the findings of this study are included within the article.
